# Effects of cognitive rehabilitation interventions on non-central nervous system cancer survivors: A meta-analysis

**DOI:** 10.1192/j.eurpsy.2023.314

**Published:** 2023-07-19

**Authors:** A. F. Oliveira, J. D. Reis, I. M. Santos, A. Torres

**Affiliations:** 1 Center for Health Technology and Services Research of the Health Research Network (CINTESIS@RISE), Department of Education and Psychology, University of Aveiro, Aveiro, Portugal; 2 Department of Mathematics, University of Aveiro, Aveiro, Portugal; 3 William James Center for Research (WJCR), Department of Education and Psychology, University of Aveiro, Aveiro, Portugal; 4 Department of Psychology and Education, Faculty of Human and Social Sciences, University of Beira Interior, Covilhã, Portugal

## Abstract

**Introduction:**

Cancer treatments can have a detrimental impact on cancer survivors’ cognitive function. Cognitive rehabilitation is considered the first-line intervention to address cognitive difficulties of cancer survivors. Nevertheless, its efficacy remains unclear.

**Objectives:**

This meta-analysis aimed to understand the effects of cognitive rehabilitation in non-central system (non-CNS) cancer survivors, through the assessment of the overall efficacy on subjective cognitive outcomes.

**Methods:**

This meta-analysis was conducted according to the Preferred Reporting Items for Systematic Review and Meta-Analysis statement. An electronic search on the databases PubMed, Scopus, and Web of Science was conducted in May 2021, considering the past 15 years, by two independent authors. Studies were eligible if they included cancer survivors (excluding CNS cancers) who were exposed to cognitive rehabilitation interventions, in which the subjective cognitive effects were measured through self-report questionnaires. The quality of studies was assessed using the Cochrane Risk of Bias Tool for Randomized Trials. The effect size was the standardized mean difference in the cognitive assessment, between baseline and post-intervention. Statistical heterogeneity was assessed using I^2^ Statistic. Publication bias was evaluated with Egger’s test. P<0.05 was considered statistically significant. The meta-analysis was performed using R software.

**Results:**

Among 14 studies, with 1115 cancer survivors, one study included a pediatric population, other young adult survivors, and the remaining adult population. The most used scale for measuring cognitive changes was the Functional Assessment of Cancer Therapy-Cognitive Function (FACT-Cog) and, as recommended, the Perceived Cognitive Impairments (PCI) subscale was used as the primary measure of subjective cognitive function. Results indicated beneficial effects following cognitive rehabilitation, with an overall standard mean difference between pre- and post-treatment of 3.4447, with CI95% [1.5543; 5.3350], p-value<0.0004. The subgroup analysis between the measures of cognitive outcomes showed that the heterogeneity is Group=Other 0.00% (I^2^) and for the Group=FACT-Cog PCI is 86% (I^2^). Analyzing the FACT-Cog PCI, the CI95% [-2.93; 6.43] includes 0, meaning that the overall effect in this subgroup is non-significant. The meta-analysis does not demonstrate publication bias (p-value of the Egger test=0.3220).

**Conclusions:**

Improvement of cognitive function in non-CNS survivors throughout cognitive rehabilitation appears to be effective. The findings of this meta-analysis can help inform clinical practice and assist practitioners in recommending and developing interventions of cognitive rehabilitation and deciding how to evaluate them. Further research is required to strengthen this evidence.

**Disclosure of Interest:**

None Declared

